# Ventriculosubgaleal shunt and neuroendoscopic lavage: refining the treatment algorithm of neonatal post-hemorrhagic hydrocephalus

**DOI:** 10.1007/s00381-021-05216-6

**Published:** 2021-05-20

**Authors:** Paolo Frassanito, Francesca Serrao, Francesca Gallini, Federico Bianchi, Luca Massimi, Giovanni Vento, Gianpiero Tamburrini

**Affiliations:** 1 Pediatric Neurosurgery, Fondazione Policlinico Universitario A. Gemelli IRCCS, Largo Agostino Gemelli, 8, 00168 Rome, Italy; 2 Neonatal Intensive Care Unit, Fondazione Policlinico Universitario A. Gemelli IRCCS, Rome, Italy; 3 Catholic University Medical School, Rome, Italy

**Keywords:** Hemorrhage, Hydrocephalus, Neuroendoscopic lavage, Personalized medicine, Preterm, Ventriculosubgaleal shunt

## Abstract

**Background:**

The optimal management of neonatal post-hemorrhagic hydrocephalus (PHH) is still debated, though several treatment options have been proposed. In the last years, ventriculosubgaleal shunt (VSgS) and neuroendosdcopic lavage (NEL) have been proposed to overcome the drawbacks of more traditional options, such as external ventricular drainage and ventricular access device.

**Methods:**

We retrospectively reviewed neonates affected by PHH treated at our institution since September 2012 to September 2020. Until 2017 patients received VSgS as initial treatment. After the introduction of NEL, this treatment option was offered to patients with large intraventricular clots. After NEL, VSgS was always placed. Primary VSgS was reserved to patients without significant intraventricular clots and critically ill patients that could not be transferred to the operating room and undergo a longer surgery.

**Results:**

We collected 63 babies (38 males and 25 females) with mean gestational age of 27.8 ± 3.8SD weeks (range 23–38.5 weeks) and mean birthweight of 1199.7 ± 690.6 SD grams (range 500–3320 g). In 6 patients, hemorrhage occurred in the third trimester of gestation, while in the remaining cases hemorrhage complicated prematurity. This group included 37 inborn and 26 outborn babies. Intraventricular hemorrhage was classified as low grade (I–II according to modified Papile grading scale) in 7 cases, while in the remaining cases the grade of hemorrhage was III to IV. Mean age at first neurosurgical procedure was 32.2 ± 3.6SD weeks (range 25.4–40 weeks). Death due to prematurity occurred in 5 patients. First-line treatment was VSgS in 49 patients and NEL in the remaining 14 cases. Mean longevity of VSgS was 30.3 days (range 10–97 days) in patients finally requiring an additional treatment of hydrocephalus. Thirty-two patients required one to three redo VSgS. Interval from initial treatment to permanent shunt ranged from 14 to 312 days (mean 70.9 days). CSF infection was observed in 5 patients (7.9%). Shunt dependency was observed in 51 out of 58 surviving patients, while 7 cases remained shunt-free at the last follow-up. Multiloculated hydrocephalus was observed in 14 cases. Among these, only one patient initially received NEL and was complicated by isolated trapped temporal horn.

**Conclusions:**

VSgS and NEL are two effective treatment options in the management of PHH. Both procedures should be part of the neurosurgical armamentarium to deal with PHH, since they offer specific advantages in selected patients. A treatment algorithm combining these two options may reduce the infectious risk and the risk of multiloculated hydrocephalus.

## Introduction

Neonatal post-hemorrhagic hydrocephalus (PHH) is a complication of intraventricular hemorrhage (IVH) in preterm babies [1] or, less frequently, of fetal intracranial hemorrhage [2]. Transient treatment of hydrocephalus is required by the presence of blood clot within the CSF and the characteristics of the preterm baby that contraindicate the placement of a permanent CSF shunting device. Several treatment options have been proposed, though the consensus on the best treatment option is still to be reached [3, 4].

In this context, ventriculo subgaleal shunt (VSgS) has emerged in the last years as a solution reducing the infectious risk and allowing to control hydrocephalus until the patient reaches an adequate body weight and the CSF clarifies [5]. More recently, neuroendoscopic lavage (NEL) has been adopted as an alternative treatment option warranting a significantly reduced risk of shunt dependency and multiloculated hydrocephalus [6–9], with good neurodevelopmental outcome [10].

We sought to develop a treatment algorithm that implements both procedures, focusing our attention on the selection of patient candidate to VSgS with or without NEL.

## Materials and method

We retrospectively collected neonatal patients affected by PHH treated at the Fondazione Policlinico Universitario A. Gemelli IRCCS, Rome, Italy, since September 2012 to September 2020. Charts were reviewed for demographic, clinical, and surgical data. Until 2017, all patients were initially treated by VSgS. After the introduction of NEL, indication to VSgS versus NEL+VSgS was carefully based on the preoperative US evaluation and clinical conditions of the baby. In fact, VSgS was indicated in cases with small intraventricular clots and preserved communication between lateral and third ventricles, warranting drainage of the ventricular system by a single ventricular catheter. On the other hand, NEL+VSgS was indicated in case of large intraventricular clots, aiming to prevent multiloculated hydrocephalus. Main contraindication to NEL+VSgS was represented by critical general conditions requiring treatment in the intensive care unit, without any possibility to transfer the patient to the surgical theater.

## Surgical technique

### VSgS

Our surgical technique for placement of VSgS has been extensively described elsewhere [11]. The procedure is performed in the surgical theater or in the intensive care unit according to the general conditions of the baby. Endotracheal intubation is not required, since the procedure lasts less than 15 min, and it is avoided whenever feasible.

**Fig. 1:**
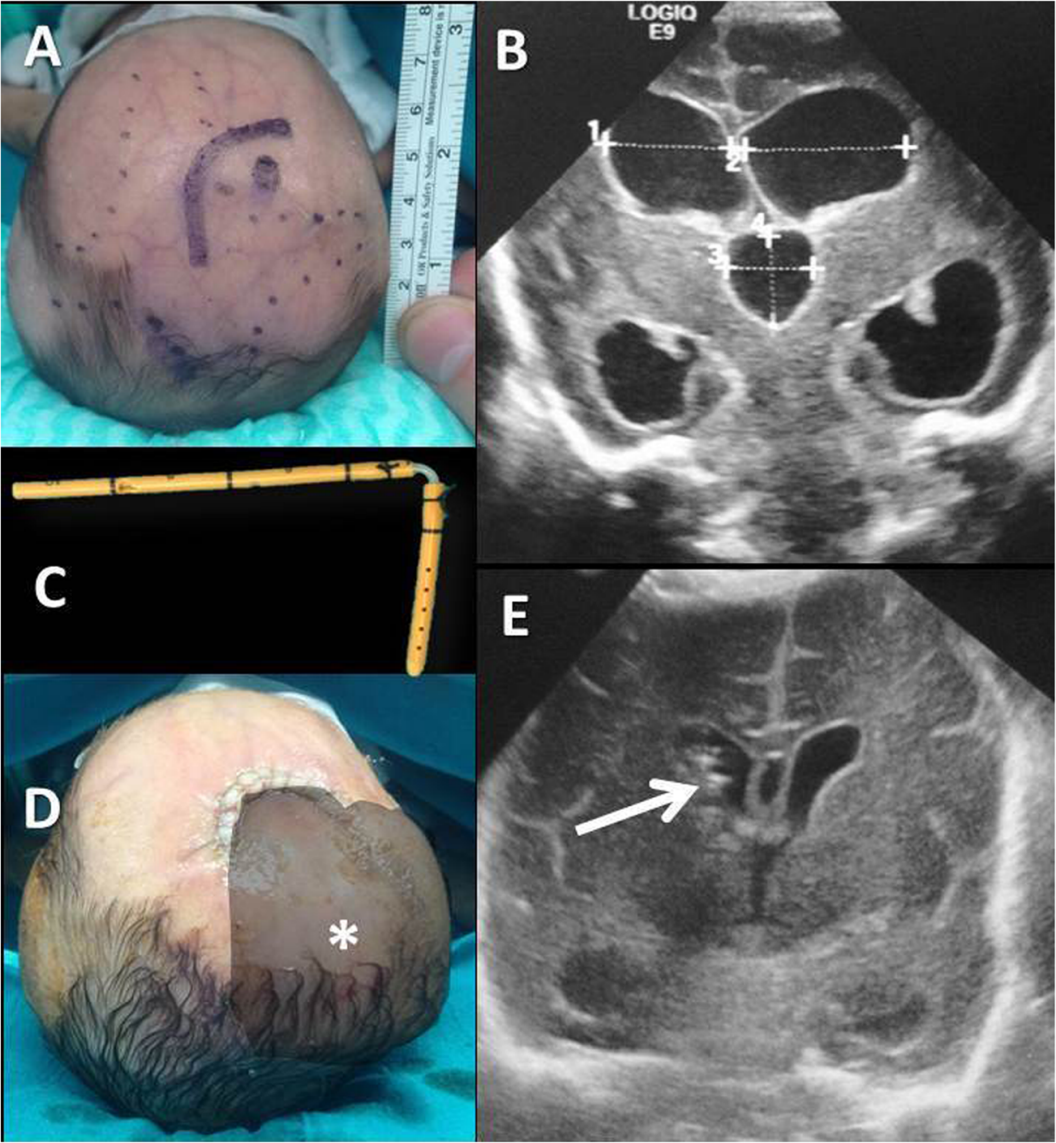
Preterm baby with large and tense anterior fontanelle and diastased sutures (A). US showing PHH without significant intraventricular clots (B). Ventriculo-subgaleal shunt harvested using an antibiotic-impregnated catheter and a right angle connector (C). Immediate postoperative picture with filled subgaleal pouch (D, shadowed area with white asterisk) and US showing correct placement of ventricular catheter (arrow) and resolution of hydrocephalus (E). (Image modified from Frassanito et al. “How to perform a ventriculo-subgaleal shunt”, Springer [11])

After careful disinfection and sterile draping, an L-shaped incision is performed in the right pre-coronaric area. This incision warrants adequate skin covering over the device, also at the time of redo VSgS, and can be easily converted in a C-shaped incision at the time of shunt conversion. A small burr hole is made using a No. 20 scalpel blade on the anterior angle of the bregmatic fontanelle, followed by dural opening and cortical coagulation. A large subgaleal pouch is harvested before ventriculostomy with blunt dissection of the subgaleal space in the ipsilateral parietal region. The device is prepared using an antibiotic-impregnated catheter (Bactiseal Codman, Integra Lifesciences, North Billerica, Billerica, MA, USA) with the interposition of a right-angle connector. The distal end is left open with additional holes in the ending segment of the tube (Fig. 1). After ventriculostomy, the device is placed and the subgaleal pouch is continuously irrigated with body-temperature saline solution to avoid the risk of abrupt drainage of the ventricles and secondary hemorrhage. Skin closure is performed with 5.0 running suture and skin glue. Transfontanellar ultrasounds (US) examination is performed to rule out immediate post-operative complications.

### NEL

The surgical technique has been extensively described in the literature [6, 7]. In the operating theater, the patient is placed supine with the head fixed in formed cotton diapers while under general anesthesia. The lateral ventricle with the largest solid hematoma is identified with transfontanellar US examination. After skin preparation, a lateralized frontal burr hole is made and the endoscope Decq (Storz, Tuttlingen, Germany) is inserted into the lateral ventricle. Irrigation of the ventricular system with 37 °C warmed Ringer solution is initiated. As appearing anatomic landmarks allowed for orientation, an interventricular septostomy is performed with tulium laser (Revolix jr, LISA laser products, Berlin, Germany) to allow irrigation of the contralateral ventricle. Solid hematoma components are aspirated by applying controlled suction with a syringe connected to the endoscopic aspiration tube, thus allowing stepwise aspiration of solid hematomas until the interface to the cerebral parenchyma was reached. The third and both lateral ventricles are cleared. Irrigation is stopped as soon as all accessible hematoma parts are aspirated and the intraventricular fluid becomes clear. After removal of the endoscope, a VSgS is placed, as previously described. The transcortical channel around the catheter is sealed with a gelatin sponge (Spongostan; Johnson & Johnson Medical, New Brunswick, NJ, USA). The sponge is designed as a ring around the vertical part of the right-angle connector, aiming to reduce the risk of intraventricular migration of the device and of the sponge [12]. The skin is meticulously closed as described above.

### Postoperative management

Postoperatively, all patients are transferred to the neonatal intensive care unit. Lateral positioning contralaterally to the subgaleal pouch is recommended, thus avoiding compression of the pouch. Clinical examination monitoring the tension of the subgaleal pouch and the head circumference is performed daily, while wound care as well as transfontanellar US are accomplished every third day. If clinical signs of active hydrocephalus reoccur, due to VSgS malfunction (collapsed subgaleal pouch or tense subgaleal pouch), conversion to permanent ventriculo-peritoneal shunt is considered if general clinical conditions are stable, patient’s weight is over 2000 g, and hemorrhage is solved on US/MR. In case of previous abdominal surgery, conversion to ventriculo-atrial shunt is indicated and it is performed if the diameter of the jugular vein is adequate and patient’s weight is over 5000 g.

Revision of VSgS is performed if patient is not suitable for conversion into permanent shunt. If VSgS is revised, the device is replaced exploiting the same frontal access and harvesting a new subgaleal pouch on the opposite side or in a site that was not dissected before. In case of conversion to permanent shunt, the shunt is placed exploiting the existing frontal access. Finally, patient may be discharged with working VSgS and monitored into outpatient clinic every other week with transfontanellar US. In case of collapsed pouch without recurrent hydrocephalus, VSgS is surgically removed, thus making the patient shunt-free.

## Results

We collected 63 babies (38 males and 25 females) with mean gestational age of 27.8 ± 3.8SD weeks (range 23–38.5 weeks) and mean birthweight of 1199.7 ± 690.6SD grams (range 500–3320 g). In 6 patients, hemorrhage occurred in the third trimester of gestation, while in the remaining cases hemorrhage complicated prematurity. This group included 37 inborn and 26 outborn babies. Intraventricular hemorrhage was classified as low grade (I–II according to modified Papile grading scale) in 7 cases, while in the remaining cases the grade of hemorrhage was III to IV. Mean age at first neurosurgical procedure was 32.2 ± 3.6SD weeks (range 25.4–40 weeks). Death due to prematurity and related complications was registered in 5 patients.

**Fig. 2:**
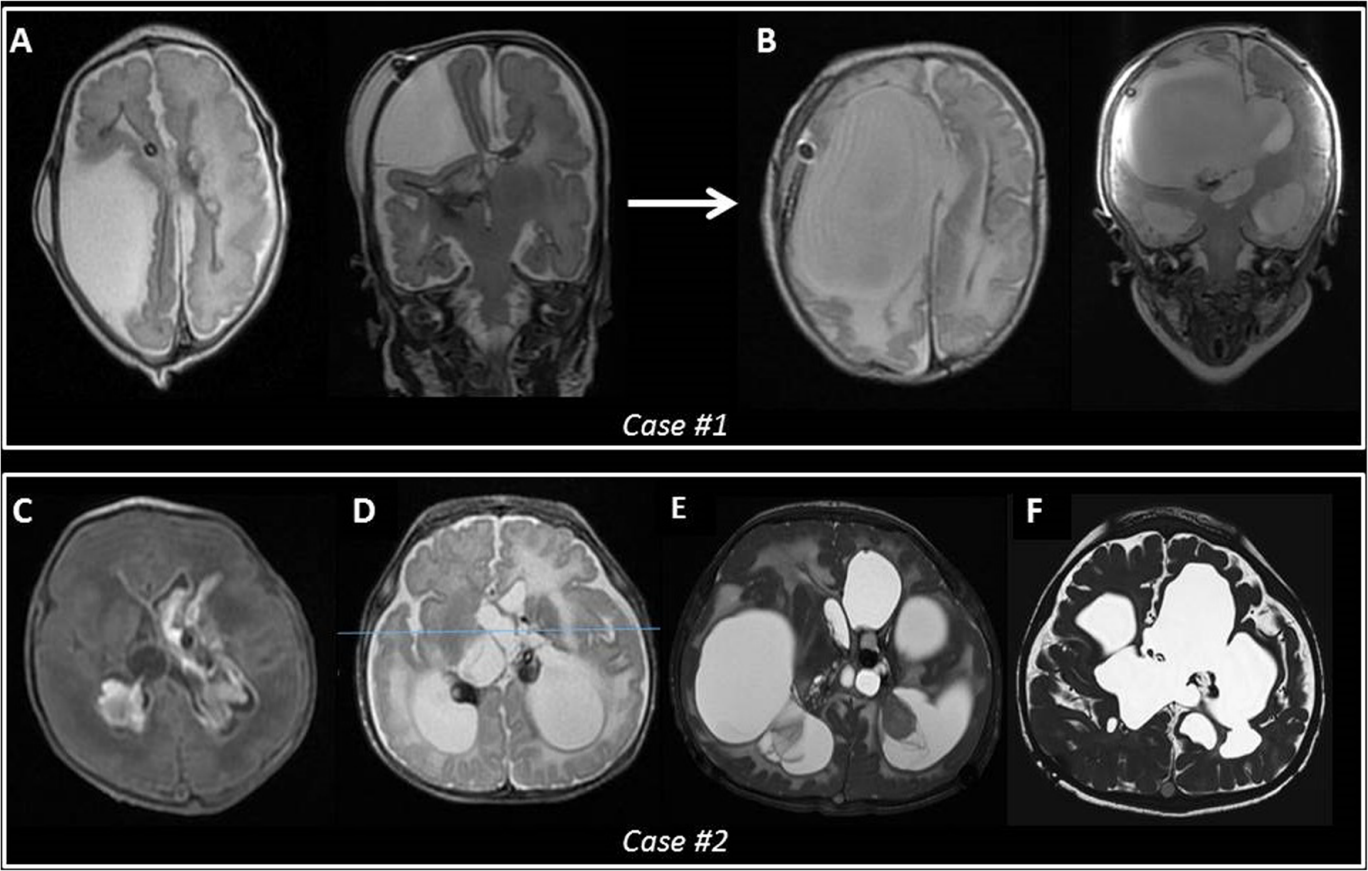
*Complications of VSgS. Case #1*—MR showing working VSgS with collapsed right ventricle and large extra-axial collection (A). Conversion of VSgS to subduro-subgaleal shunt allowed progressive re-expansion of the ventricle and drainage of the subdural hygroma (B). *Case #2***—**Preterm intraventricular hemorrhage, causing hydrocephalus that was treated by VSgS (C), before the introduction of NEL, and complicated by bilateral entrapment of ventricular horn (D), that required several additional endoscopic procedures with CSF leak and infection leading to multiloculated hydrocephalus (E). Last follow-up MR after endoscopic fenestration of intraventricular septa and VP shunt (F)

First-line treatment was VSgS in 49 patients and NEL in the remaining 14 cases. Mean time of VSgS was 30.3 days, ranging from 10 to 97 days in patients finally requiring an additional treatment of hydrocephalus. Thirty-two patients required one to three redo VSgS. Additional treatments in patients receiving VSgS as first treatment were NEL in 4 cases, EVD in 9 cases, and other endoscopic procedures for fenestration of intraventricular septa in 3 cases. One patient was complicated by subdural hygroma, requiring conversion of the VSgS into a subduro-subgaleal shunt (Fig. 2A–B). One patient was complicated by symptomatic upward transtentorial herniation at the time of redo VSgS, treated by a second VSgS draining the trapped fourth ventricle.

Time from initial treatment to permanent shunt ranged from 14 to 312 days (mean 70.9 days). Interestingly, 20 patients were discharged with VSgS.

CSF infection was observed in 5 patients (7.9%). In particular, infection finally complicated CSF leak in three cases who underwent several procedures for treatment of multiloculated hydrocephalus before the introduction of NEL (Fig. 2C–F). In the remaining two cases, CSF infection was secondary to sepsis resulting from severe necrotizing enterocolitis.

Shunt dependency was observed in most of the cases with conversion to ventriculo-peritoneal shunt in 43 cases and ventriculo-atrial shunt in 8 cases. Seven cases remained shunt-free at the latest follow-up; two of them received ETV at the time of VSgS removal.

Multiloculated hydrocephalus was observed in 14 cases, requiring isolated aqueductoplasty in 4 cases, septostomy in 2 cases, fenestration of trapped temporal horn in 2 cases, and fenestration of multiple intraventricular septa/cysts in the 6 remaining cases. Among these, only one patient initially received NEL and was complicated by isolated trapped temporal horn.

With regard to the implementation of NEL in our treatment algorithm, thirty-nine patients were treated in the first part of our experience (VSgS) and 24 after the introduction of the new protocol (VSgS ± NEL). Results are presented in Table 1.

**Table 1:** Characteristics of patients treated with VSgS (2012–2017) or VSgS ± NEL (2018–2020)

	Total	VSgS	VSgS ± NEL
Patients	63	39	24
GA (range)	27.8 ± 3.8SD weeks (23–38.5 w)	27.6 ± 4SD weeks (23–38.5w)	28.7 ± 3.4SD weeks (25–38.5w)
Weight (range)	1199.7 ± 690.6SD grams (500-3320 g)	1147.3 ± 675.2SD grams (530–3320 g)	1306 ± 687.2SD grams (500–3135 g)
GA at first surgery (range)	32.2 ± 3.6SD weeks (25.4–40 w)	32 ± 3.8SD weeks (25.4–40w)	32.7 ± 3.2SD weeks (28.1–38.3w)
Infection	5 (7.9%)	4 (10.3%)	1 (4.2%)
Multiloculated hydrocephalus	14 (22.2%)	9 (23.1%)	5 (20.8%)
Multiple intraventricular septations	6 (9.5%)	5 (12.8%)	1 (4.2%)
Death	5 (7.9%)	4 (10.3%)	1 (4.2%)
Shunt-free	7	4	3
Shunt dependency	87.9%	88.6%	87%

## Discussion

The treatment of neonatal PHH is still challenging due to the fragility of patients and other critical factors. Indeed, features of either the patient, namely prematurity and low weight, and the CSF, such as the blood clots and high protein concentration, and the receiving site of extrathecal CSF shunting, that are necrotizing enterocolitis for the abdomen and inadequate caliber of jugular vein in case of ventriculo-atrial shunt, require transient treatment of hydrocephalus, thus delaying the timing of permanent shunt until the previous conditions are solved.

External ventricular drainage (EVD) may be inserted at bedside, theoretically warrants continuous CSF drainage, and can be easily removed [13]. These advantages are counterbalanced by the ineluctable infectious risk. Additionally, adequate subcutaneous tunnel could be not performed, subcutaneous tissue is loose and poor, and the skin is loose, these factors favoring CSF leak and pullout of the catheter. Other drawbacks are the low-pressure drainage, the frequent obstruction of the catheter by clot, and the presence of multiple lines that complicate the management of the patient and increase the risk of accidental pullout. Finally, risks of overdrainage and hyponatremia are not negligible. Antibiotic-impregnated catheter [14] and subcutaneous sutureless device have been implemented in our practice to reduce the risk of EVD [15, 16]. However, mean time to conversion to permanent shunt is longer than 2 months in the present series, thus suggesting the cumulative risk of relying on EVD for such a long time.

On these grounds, we reserve EVD to patients with infection of CSF or blood, who could not receive VSgS.

Ventricular access device (VAD) partly overcomes limits of EVD [17, 18], but repeated tapping of the subcutaneous reservoir is burdened by ineluctable risk of infection and raises concerns on the alternate control of raised intracranial pressure.

These considerations prompted us to adopt VSgS in the management of PHH. Indeed, this treatment option proved to be effective and safe. Main advantages of VsgS are avoiding the risk of electrolyte imbalance secondary to CSF drainage, the easiness to manage the patient by the nursing and medical staff after adequate education, and the possibility to discharge the patient to home before permanent treatment of hydrocephalus. Furthermore, the length of the procedure is usually less than 15 min and could be performed at the bedside.

Infectious risk seems lower compared to other treatment options, namely EVD and VAD. It is of worth to note that series of VSgS reporting no infection include less than 20 patients [19, 20], while the risk of infection is constant in larger series [3, 21–23] and ranges from 8 to 10% in series with more than fifty patients [24, 25] (Table 2).

**Table 2:** Synopsis of series including more than 10 patients affected by PHH managed by ventriculo-subgaleal shunt or neuroendoscopic lavage

Reference	VSgS *(Technical details)*			PHH cases	Mean body weight (g)	Mean gestational age (weeks)	Infections (%)	Mean duration of VSgS (days)	Mortality (%)	Shunt dependency in surviving patients (%)
	***SubQ reservoir***	***Distal end***	***Other***							
Sklar et al. 1992	Low pressure Pudenz pumping device (Heyer-Schulte)	Low-pressure peritonel anti-reflux catheter	Shunt pumping if small subgaleal collection	62	1560	29.8	10	n.s	11% *(1.6% related to shunt procedure*)	89.1
Rahman et al. 1995	Low pressure Pudenz pumping device (Heyer-Schulte)	Open	Daily shunt pumping if small subgaleal collection. Tapping if tense subgaleal collection	15	1284	29	0	9.2 weeks	0	80
Karas et al. 2007	No	Peritoneal anti-reflux catheter		17	n.s.	26	0	56.1	0	70.6
Lam et al. 2009	n.s.	Open	Tapping if tense subgaleal collection	16	998	27.4	6.3	n.s.	12.5	71.4
Limbrick et al. 2010	McComb reservoir	-Open (4 cases), -Medium-pressure (21 cases), Low-pressure (5 cases) distal slit valve (Vygon Neuro)	Tapping if tense subgaleal collection	30	n.s.	31.7	3.3	20 weeks	13.3	76.9
Koksal and Oktem 2010	No	Open	n.s.	25	1342	29.3	8	44	28	83.3
Nagy et al. 2013	Ventricular catheter with integrated reservoir (Sophysa, PRO6)	Open	Tapping if tense subgaleal collection	72	1037	27.3	8.3	87.9	4.2	100
Ellenbogen et al. 2016	n.s	n.s.	n.s.	22	n.s.	27	9	n.s.	n.s.	73
Etus et al. 2018	n.s.	n.s.	n.s.	22	<1500	n.s.	36.6	n.s.	n.s.	77.2
	**NEL** **Reservoir** **Additional measure** **Relavage**			**PHH cases**	**Mean body weight (g)**	**Mean gestational age (weeks)**	**Infections (%)**		**Mortality**	**Shunt dependency in surviving patients (%)**
	**Additional Measure**	**Relavage**								
Schulz et al. 2014	SubQ reservoir			19	1036	27.8	0		0	58
Etus et al. 2018	EVD			23	<1500	n.s.	4.3		n.s.	60.8
D’Arcangues et al 2018	SubQ reservoir/EVD			56	1523	31.3	3.6		5.4	58.5
Tirado-caballero et al. 2020	No subQ reservoir	Yes		46	1671.86	30.04	21.7		6.5 (in the first year)	58.7
	**VSgS ± NEL** ***(Techniqual details)***			**PHH cases**	**Mean body weight (g)**	**Mean gestational age (weeks)**	**Infections (%)**	**Mean duration of VSgS (days)**	**Mortality (%)**	**Shunt dependency in surviving patients (%)**
	***SubQ reservoir***	***Distal end***	***Other***							
*Present study*	*No*	*Open*	*ATB-impregnated catheter* *No tapping policy*	*63*	*1200*	*27.8*	*7.9*	*32.2*	*7.9*	*87.9*

*ATB*, antibiotic; *NEL*, neuroendoscopic lavage; *n.s.*, not stated; *PHH*, post-hemorrhagic hydrocephalus; *SubQ*, subcutaneous; *VSgS*, ventriculo-subgaleal shunt

Other complications are hemorrhage, due to abrupt drainage of ventricular system, and subdural hygroma hematoma.

Skin dehiscence with device exposure and CSF leak may be secondary to loose skin. Additionally, VSgS shares mechanical complications, such as obstruction and migration, with other shunt devices [26].

Technical aspects of VSgS are not standardized through the literature, in particular concerning the use of valve versus valveless tube and the use of reservoir. The use of valve may reduce the risk of abrupt drainage of the ventricular system and related complications, but it is associated to a major risk of obstruction by blood clot and collapsed subgaleal pouch. On these grounds, daily pumping is recommended by centers using VSgS with valve [20, 24].

Additionally, the management of VSgS when subgaleal pouch is satured varies across the literature. In the majority of experiences, tapping of the pouch or of the reservoir is performed to extend the life of the VSgS. This somehow converts the VSgS into a ventricular access device and may affect the comparison of morbidity between the two options [23].

In our experience, we designed a bundle exploiting antibiotic-impregnated catheter and avoiding to tap the device or the pouch aiming to reduce infectious risk. Indeed, infection is the only factor, along with entity of brain damage by the initial hemorrhage, affecting the neurocognitive outcome of these patients [27]. Additionally, CSF infection critically contributes to the occurrence of complex forms of multiloculated hydrocephalus, thus furtherly complicating the management of PHH [28, 29].

Our management protocol of VSgS warranted an acceptable risk of infection, though this risk is ineluctable as the device and CSF could be infected secondarily to blood sepsis.

With the present no-tapping policy, the mean life expectancy of VSgS is about 32 days and it could be not compared to other experiences using daily tapping to extend the life of VSgS after saturation of the subgaleal pouch.

Other complications, secondary to abrupt drainage of the ventricular system, such as subdural hygroma and intraventricular hemorrhage occurred in the initial phase of this experience.

Some surgical nuances, as accurate choice of the side of ventriculostomy avoiding area with very thin brain parenchymal thickness and continuous irrigation of the subgaleal pouch during skin closure, helped to prevent these complications.

This retrospective analysis revealed three cases complicated by primary CSF infection without general sepsis before the introduction of NEL. Risk factors were several surgical and endoscopic procedures for multiloculated hydrocephalus and CSF leak. Indeed, the presence of large intraventricular clots favored a bilateral entrapment of temporal horns.

**Fig. 3:**
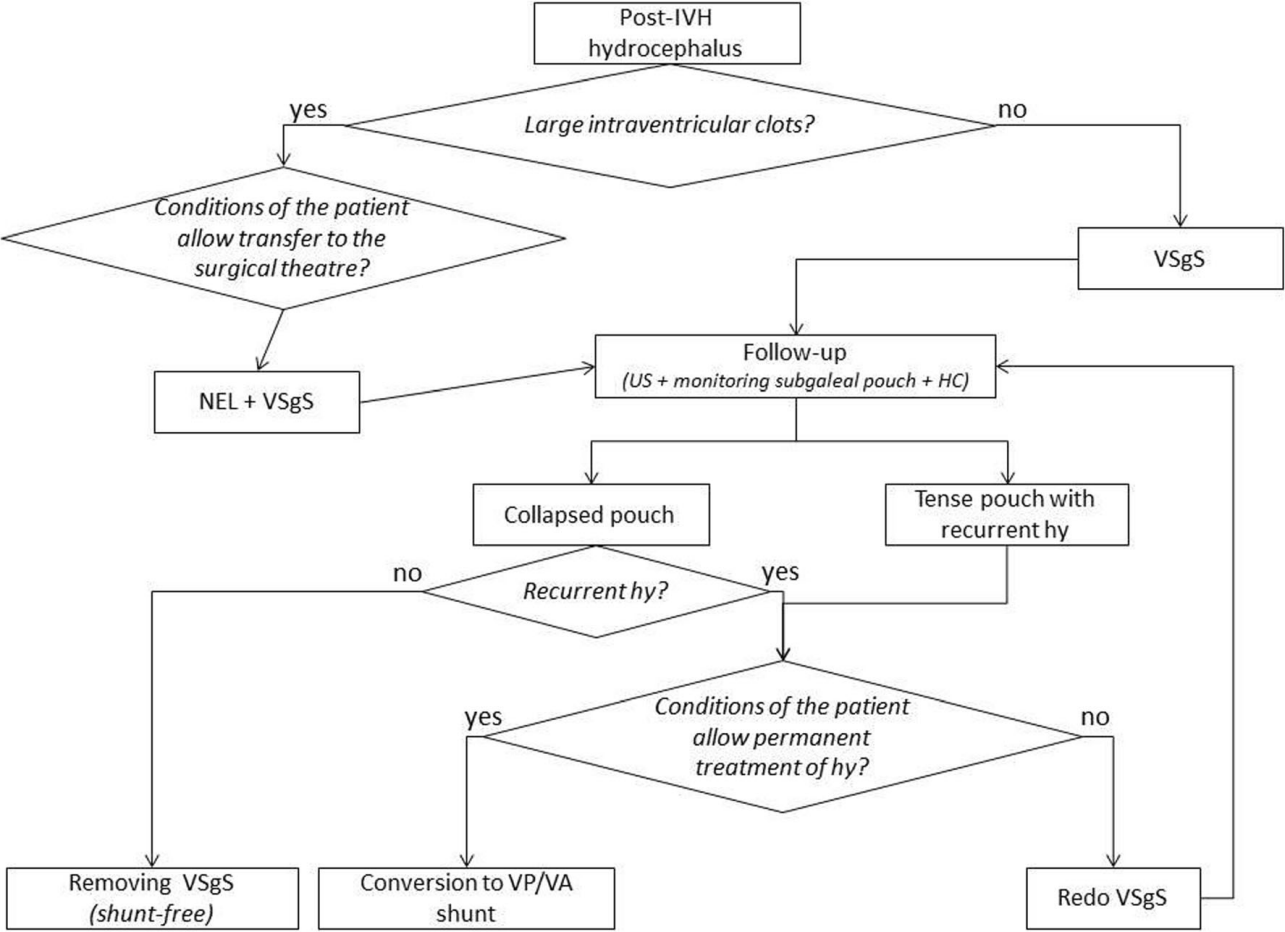
Management algorithm of post-intraventricular hemorrhage (IVH) hydrocephalus

On these grounds, we implemented NEL in our management algorithm of PHH to prevent multiloculated complications and related morbidity (Fig. 3), as supported by literature data [6].

These treatment options have been proposed as alternative options by different neurosurgical centers. However, VSgS and NEL may have different indications and should be both available in the surgical armamentarium of pediatric neurosurgeons.

VSgS may be placed in the intensive care unit [19], thus representing an effective option for patients with critical clinical conditions contraindicating the transfer to the surgical theater and a longer surgical procedure, such as NEL. In our experience, this condition was usually observed in inborn patients with lower birthweight and more severe prematurity. Furthermore, VSgS may effectively control hydrocephalus in patients with xantocromic CSF without large intraventricular clots. Conversely, we observed this condition in particular in outborn patients, who were transferred to our institution after stabilization of clinical conditions and were treated at elder age compared to inborn patients. In these cases, treatment of PHH was easier due to spontaneous resolution of blood clots inside the ventricles and elder age of the baby at treatment, though this condition has been recently associated to worse outcome [30].

**Fig. 4:**
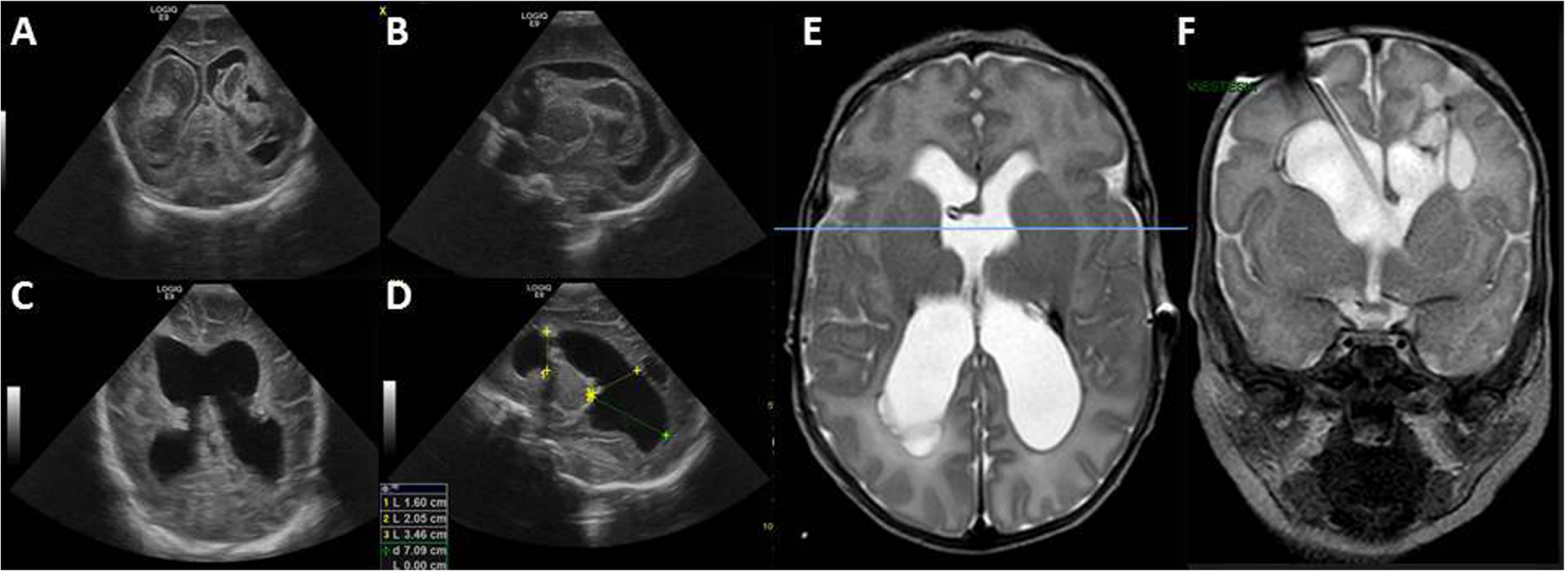
Preoperative transfontanellar US showing PHH with large intraventricular clots (A, B) that were completely removed with NEL, as confirmed by postoperative US (C, D). MR after conversion of VSgS to VP shunt ruling out multiloculated hydrocephalus (E, F)

On the other side, NEL should be performed in the surgical theater and it reduces the risk of multiloculated hydrocephalus in patients with large intraventricular clots (Fig. 4). This treatment option is offered to babies with stable general conditions.

Literature data also show that NEL could significantly reduce the rate of shunt dependency [6]. However, this effect was not confirmed by the present experience, showing a rate of shunt dependency over 80%, similarly to previous series of VSgS, and without significant difference between patients undergoing NEL and patients undergoing VSgS as first treatment. Further studies with larger numbers are advocated to analyze the impact of other factors, such as the selection of patients and timing of surgery on this outcome measure.

In conclusion, this study represents the first institutional experience combining VSgS and NEL in the management of PHH. Although longer follow-up is required to study the neurocognitive outcome of these patients [10], these data would be essential to discuss the results of the ongoing TROPHY registry study, aiming to finally define the best management of PHH and standardize its treatment [4].

## Conclusions

VSgS and NEL are two effective treatment options in the management of PHH. Both procedures should be part of the neurosurgical armamentarium to deal with PHH, since they offer specific advantages in selected patients. A treatment algorithm combining these two options may reduce the infectious risk and the risk of multiloculated hydrocephalus.
